# 1-(3-Ethyl­phen­yl)-4,6-dimethyl-2-oxo-1,2-dihydro­pyridine-3-carbonitrile

**DOI:** 10.1107/S1600536812019927

**Published:** 2012-05-12

**Authors:** Mansour S. Al-Said, Mostafa M. Ghorab, Hazem A. Ghabbour, Suhana Arshad, Hoong-Kun Fun

**Affiliations:** a Medicinal, Aromatic and Poisonous Plants Research Center (MAPPRC), College of Pharmacy, King Saud University, PO Box 2457, Riyadh 11451, Saudi Arabia; b Department of Pharmaceutical Chemistry, College of Pharmacy, King Saud University, PO Box 2457, Riyadh 11451, Saudi Arabia; c X-ray Crystallography Unit, School of Physics, Universiti Sains Malaysia, 11800 USM, Penang, Malaysia

## Abstract

In the title compound, C_16_H_16_N_2_O, the essentially planar 1,2-dihydro­pyridine ring [maximum deviation = 0.021 (1) Å] makes a dihedral angle of 85.33 (8)° with the benzene ring. In the crystal, mol­ecules are linked into a chain along the *b* axis *via* C—H⋯O inter­actions.

## Related literature

For the biological activities and applications of 2-pyridone derivatives, see: Abadi *et al.* (2009 ▶); Cheney *et al.* (2007 ▶); Aqui & Vonderheide (2008 ▶); Ambrosini *et al.* (1997 ▶); Murata *et al.* (2001 ▶); Ghorab *et al.* (2009 ▶, 2010 ▶); Al-Said *et al.* (2010 ▶). For related structures, see: Lynch & McClenaghan (2002 ▶); Elgemeie & Jones (2004 ▶).
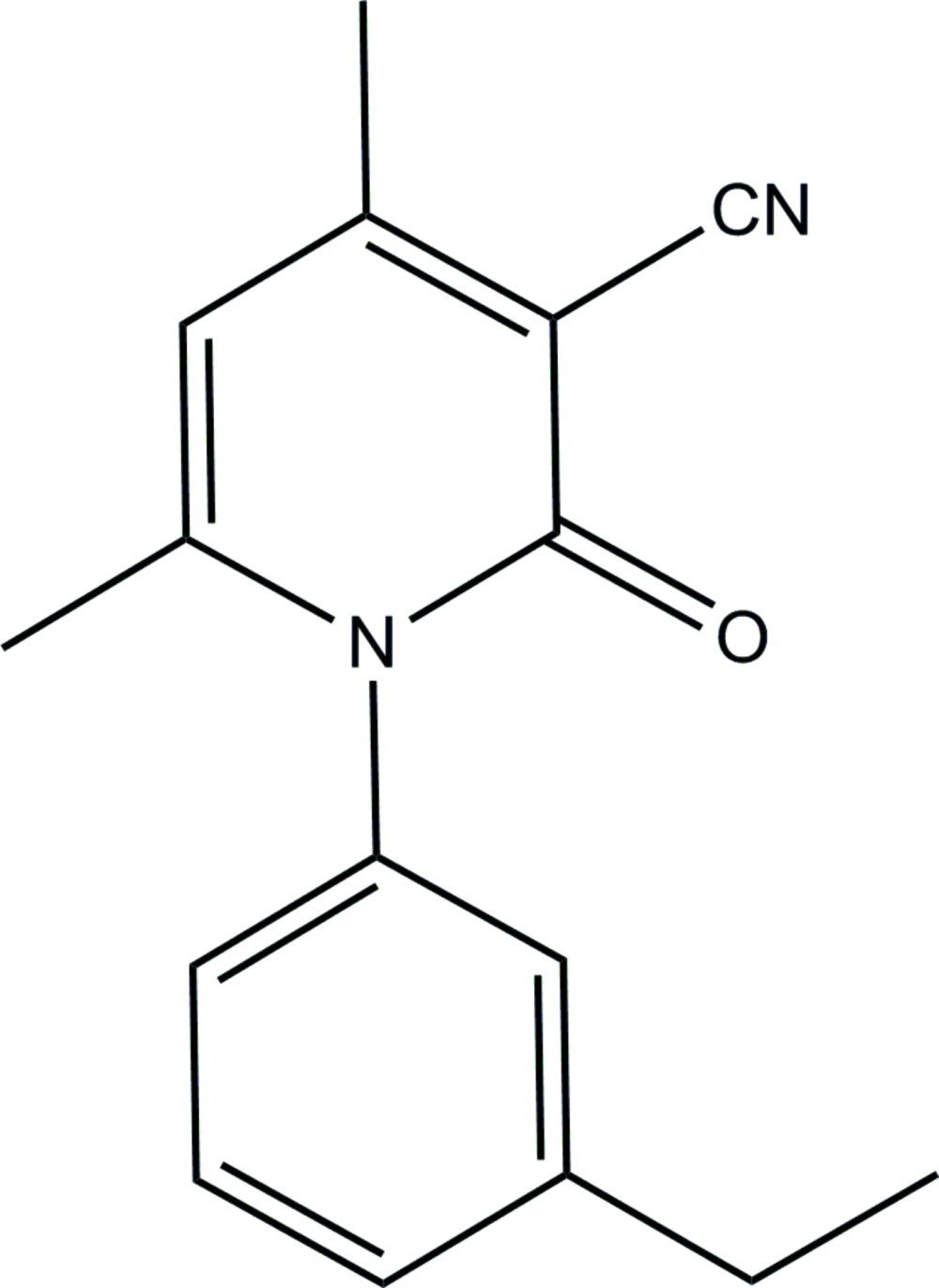


## Experimental

### 

#### Crystal data

C_16_H_16_N_2_O
*M* *_r_* = 252.31
Monoclinic, 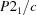

*a* = 8.3834 (3) Å
*b* = 7.1852 (2) Å
*c* = 23.5264 (8) Å
β = 93.203 (3)°
*V* = 1414.93 (8) Å^3^
*Z* = 4
Cu *K*α radiation
μ = 0.59 mm^−1^
*T* = 296 K
0.93 × 0.46 × 0.07 mm

#### Data collection

Bruker APEXII CCD diffractometer
Absorption correction: multi-scan (*SADABS*; Bruker, 2009 ▶) *T*_min_ = 0.609, *T*_max_ = 0.960
9742 measured reflections
2638 independent reflections
1938 reflections with *I* > 2σ(*I*)
*R*_int_ = 0.032

#### Refinement

*R*[*F* ^2^ > 2σ(*F* ^2^)] = 0.047
*wR*(*F* ^2^) = 0.161
*S* = 1.09
2638 reflections
170 parameters
H-atom parameters constrained
Δρ_max_ = 0.30 e Å^−3^
Δρ_min_ = −0.18 e Å^−3^

### 

Data collection: *APEX2* (Bruker, 2009 ▶); cell refinement: *SAINT* (Bruker, 2009 ▶); data reduction: *SAINT*; program(s) used to solve structure: *SHELXTL* (Sheldrick, 2008 ▶); program(s) used to refine structure: *SHELXTL*; molecular graphics: *SHELXTL*; software used to prepare material for publication: *SHELXTL* and *PLATON* (Spek, 2009 ▶).

## Supplementary Material

Crystal structure: contains datablock(s) global, I. DOI: 10.1107/S1600536812019927/is5134sup1.cif

Structure factors: contains datablock(s) I. DOI: 10.1107/S1600536812019927/is5134Isup2.hkl

Supplementary material file. DOI: 10.1107/S1600536812019927/is5134Isup3.cml

Additional supplementary materials:  crystallographic information; 3D view; checkCIF report

## Figures and Tables

**Table 1:** Hydrogen-bond geometry (Å, °)

*D*—H⋯*A*	*D*—H	H⋯*A*	*D*⋯*A*	*D*—H⋯*A*
C4—H4*A*⋯O1^i^	0.93	2.32	3.2105 (18)	161

Symmetry code: (i) 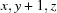.

**Fig. 1.**
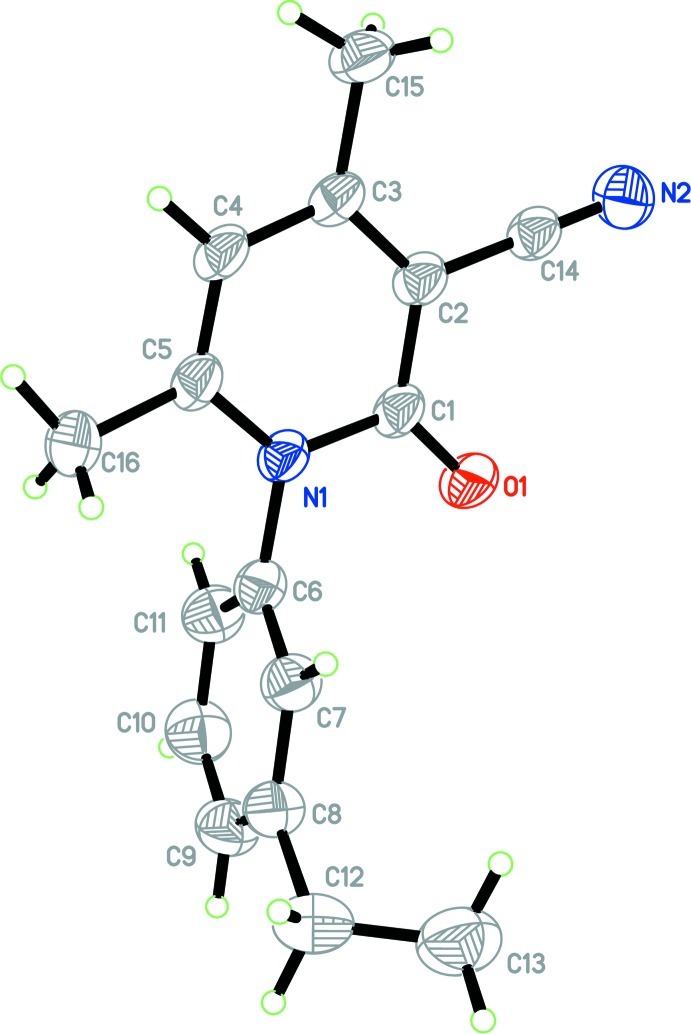
The molecular structure of the title compound, showing 30% probability displacement ellipsoids and the atom-numbering scheme.

**Fig. 2.**
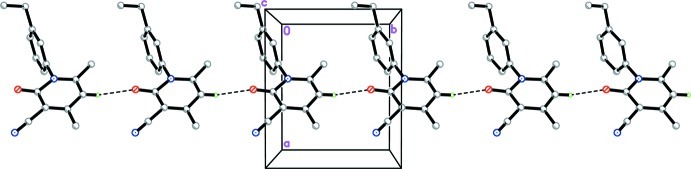
A crystal packing diagram of the title compound viewed along the c axis. For the sake of clarity, H atoms not involved in the intermolecular interactions (dashed lines) have been omitted.

## supplementary crystallographic information

### Comment

It was reported that compounds containing the 2-pyridone moiety was proven to possess several biological properties, especially anticancer activity (Abadi *et al.*, 2009; Cheney *et al.*, 2007; Aqui & Vonderheide, 2008; Ambrosini *et al.*, 1997; Murata *et al.*, 2001). Compounds containing heteroaromatic rings frequently play an important role as scaffolds of bioactive substances. It is well known that pyridone and its derivatives are among the most popular *N*-heteroaromatic compounds integrated into the structures of many pharmaceutical compounds and their structural units occur in various molecules exhibiting diverse biological activities (Abadi *et al.*, 2009). Based on the above information and as a continuation of our previous work on anticancer agents (Ghorab *et al.*, 2009; Al-Said *et al.*, 2010; Ghorab *et al.*, 2010), we report the synthesis of a novel 2-pyridone derivative which is expected to exhibit anticancer activity.

In the title compound (Fig. 1), the 1,2-dihydropyridine ring (N1/C1–C5) is essentially planar with a maximum deviation of 0.021 (1) Å at atom N1 and almost perpendicular with the benzene ring (C6–C11) with a dihedral angle of 85.33 (8)°. Bond lengths and angles are within the normal ranges and are comparable to those in the related structures (Lynch & McClenaghan, 2002; Elgemeie & Jones, 2004). The crystal structure is shown in Fig. 2. The molecules are linked into a one-dimensional chain along the *b*-axis *via* C4—H4A···O1 interactions (Table 1).

### Experimental

A mixture of 2-cyano-*N*-(3-ethylphenyl)acetamide (1.88 g, 0.01 mol) and acetylacetone (1.00 g, 0.01 mol) in absolute ethanol (50 ml) containing piperidine (0.5 ml) were refluxed for 5 h. The reaction mixture was triturated with ethanol and the solid obtained was recrystallized from ethanol to give the title compound. A single-crystal suitable for an X-ray structural analysis was obtained by slow evaporation from an ethanol solution at room temperature.

### Refinement

All H atoms were positioned geometrically (C—H = 0.93–0.97 Å) and refined using a riding model, with *U*_iso_(H) = 1.2 or 1.5*U*_eq_(C). A rotating group model was applied to the methyl groups. The same *U^ij^* parameter was used for atoms pair C2/C14.

### Crystal data

**Table:**

C_16_H_16_N_2_O	*F*(000) = 536
*M**_r_* = 252.31	*D*_x_ = 1.184 Mg m^−^^3^
Monoclinic, *P*2_1_/*c*	Cu *K*α radiation, λ = 1.54178 Å
Hall symbol: -P 2ybc	Cell parameters from 1728 reflections
*a* = 8.3834 (3) Å	θ = 3.8–62.6°
*b* = 7.1852 (2) Å	µ = 0.59 mm^−^^1^
*c* = 23.5264 (8) Å	*T* = 296 K
β = 93.203 (3)°	Plate, colorless
*V* = 1414.93 (8) Å^3^	0.93 × 0.46 × 0.07 mm
*Z* = 4	

### Data collection

**Table:**

Bruker APEXII CCD diffractometer	2638 independent reflections
Radiation source: fine-focus sealed tube	1938 reflections with *I* > 2σ(*I*)
Graphite monochromator	*R*_int_ = 0.032
φ and ω scans	θ_max_ = 69.8°, θ_min_ = 3.8°
Absorption correction: multi-scan (*SADABS*; Bruker, 2009)	*h* = −10→10
*T*_min_ = 0.609, *T*_max_ = 0.960	*k* = −6→8
9742 measured reflections	*l* = −28→28

### Refinement

**Table:**

Refinement on *F*^2^	Secondary atom site location: difference Fourier map
Least-squares matrix: full	Hydrogen site location: inferred from neighbouring sites
*R*[*F*^2^ > 2σ(*F*^2^)] = 0.047	H-atom parameters constrained
*wR*(*F*^2^) = 0.161	*w* = 1/[σ^2^(*F*_o_^2^) + (0.0972*P*)^2^ + 0.0312*P*] where *P* = (*F*_o_^2^ + 2*F*_c_^2^)/3
*S* = 1.09	(Δ/σ)_max_ < 0.001
2638 reflections	Δρ_max_ = 0.30 e Å^−^^3^
170 parameters	Δρ_min_ = −0.18 e Å^−^^3^
0 restraints	Extinction correction: *SHELXL*, Fc^*^=kFc[1+0.001xFc^2^λ^3^/sin(2θ)]^-1/4^
Primary atom site location: structure-invariant direct methods	Extinction coefficient: 0.0026 (7)

### Special details

**Table:**

Geometry. All e.s.d.'s (except the e.s.d. in the dihedral angle between two l.s. planes) are estimated using the full covariance matrix. The cell e.s.d.'s are taken into account individually in the estimation of e.s.d.'s in distances, angles and torsion angles; correlations between e.s.d.'s in cell parameters are only used when they are defined by crystal symmetry. An approximate (isotropic) treatment of cell e.s.d.'s is used for estimating e.s.d.'s involving l.s. planes.
Refinement. Refinement of *F*^2^ against ALL reflections. The weighted *R*-factor *wR* and goodness of fit *S* are based on *F*^2^, conventional *R*-factors *R* are based on *F*, with *F* set to zero for negative *F*^2^. The threshold expression of *F*^2^ > σ(*F*^2^) is used only for calculating *R*-factors(gt) *etc*. and is not relevant to the choice of reflections for refinement. *R*-factors based on *F*^2^ are statistically about twice as large as those based on *F*, and *R*- factors based on ALL data will be even larger.

### Fractional atomic coordinates and isotropic or equivalent isotropic displacement parameters (Å^2^)

**Table:**

	*x*	*y*	*z*	*U*_iso_*/*U*_eq_	
O1	0.51182 (17)	−0.19353 (15)	0.40553 (6)	0.0771 (4)	
N1	0.43401 (15)	0.09675 (17)	0.37682 (6)	0.0565 (4)	
N2	0.8174 (2)	−0.1541 (2)	0.51433 (8)	0.0818 (5)	
C1	0.52879 (18)	−0.0253 (2)	0.41097 (7)	0.0567 (4)	
C2	0.64093 (18)	0.0642 (2)	0.45059 (7)	0.0596 (3)	
C3	0.65232 (18)	0.2536 (2)	0.45604 (7)	0.0574 (4)	
C4	0.54774 (19)	0.3644 (2)	0.42144 (8)	0.0614 (4)	
H4A	0.5523	0.4932	0.4250	0.074*	
C5	0.44005 (19)	0.2869 (2)	0.38292 (7)	0.0580 (4)	
C6	0.32547 (19)	0.0076 (2)	0.33530 (7)	0.0599 (4)	
C7	0.1715 (2)	−0.0320 (2)	0.34863 (8)	0.0687 (5)	
H7A	0.1361	0.0037	0.3838	0.082*	
C8	0.0682 (2)	−0.1252 (3)	0.30994 (9)	0.0756 (5)	
C9	0.1242 (3)	−0.1759 (3)	0.25855 (9)	0.0826 (6)	
H9A	0.0571	−0.2395	0.2324	0.099*	
C11	0.3787 (2)	−0.0421 (3)	0.28313 (8)	0.0763 (5)	
H11A	0.4822	−0.0133	0.2739	0.092*	
C12	−0.0998 (3)	−0.1725 (4)	0.32586 (12)	0.1040 (8)	
H12A	−0.1475	−0.0635	0.3423	0.125*	
H12B	−0.1637	−0.2053	0.2917	0.125*	
C13	−0.1029 (3)	−0.3299 (4)	0.36728 (13)	0.1134 (9)	
H13A	−0.2115	−0.3566	0.3754	0.170*	
H13B	−0.0437	−0.2960	0.4018	0.170*	
H13C	−0.0556	−0.4381	0.3512	0.170*	
C14	0.73956 (19)	−0.0576 (2)	0.48568 (7)	0.0596 (3)	
C15	0.7695 (2)	0.3428 (2)	0.49802 (9)	0.0739 (5)	
H15A	0.8759	0.3053	0.4901	0.111*	
H15B	0.7470	0.3047	0.5358	0.111*	
H15C	0.7607	0.4757	0.4950	0.111*	
C16	0.3267 (2)	0.4026 (3)	0.34699 (9)	0.0788 (6)	
H16A	0.3524	0.5319	0.3524	0.118*	
H16B	0.2196	0.3803	0.3578	0.118*	
H16C	0.3350	0.3703	0.3077	0.118*	
C10	0.2776 (3)	−0.1347 (3)	0.24493 (9)	0.0876 (6)	
H10A	0.3129	−0.1697	0.2097	0.105*	

### Atomic displacement parameters (Å^2^)

**Table:**

	*U*^11^	*U*^22^	*U*^33^	*U*^12^	*U*^13^	*U*^23^
O1	0.0885 (9)	0.0349 (6)	0.1054 (10)	−0.0021 (5)	−0.0179 (7)	−0.0017 (5)
N1	0.0590 (7)	0.0398 (7)	0.0703 (8)	−0.0013 (5)	0.0004 (6)	0.0012 (5)
N2	0.0869 (11)	0.0633 (10)	0.0934 (12)	0.0139 (8)	−0.0113 (9)	0.0020 (7)
C1	0.0609 (8)	0.0358 (8)	0.0735 (10)	0.0006 (6)	0.0040 (7)	0.0010 (6)
C2	0.0608 (6)	0.0431 (6)	0.0747 (7)	0.0014 (5)	0.0019 (5)	−0.0009 (5)
C3	0.0564 (8)	0.0408 (8)	0.0754 (10)	−0.0031 (6)	0.0078 (7)	−0.0021 (6)
C4	0.0657 (9)	0.0343 (7)	0.0842 (11)	−0.0015 (6)	0.0040 (8)	−0.0001 (7)
C5	0.0611 (9)	0.0393 (8)	0.0740 (10)	0.0006 (6)	0.0066 (7)	0.0047 (6)
C6	0.0643 (9)	0.0441 (8)	0.0708 (10)	0.0003 (7)	−0.0015 (7)	0.0005 (6)
C7	0.0668 (9)	0.0638 (11)	0.0751 (11)	−0.0024 (8)	0.0006 (8)	−0.0010 (8)
C8	0.0698 (10)	0.0683 (11)	0.0872 (13)	−0.0069 (9)	−0.0094 (9)	0.0029 (9)
C9	0.0881 (14)	0.0756 (13)	0.0816 (13)	−0.0042 (9)	−0.0162 (11)	−0.0080 (9)
C11	0.0761 (11)	0.0758 (13)	0.0772 (12)	−0.0043 (9)	0.0074 (9)	−0.0066 (9)
C12	0.0726 (13)	0.117 (2)	0.120 (2)	−0.0191 (12)	−0.0096 (12)	−0.0005 (15)
C13	0.0886 (15)	0.143 (3)	0.1091 (19)	−0.0278 (15)	0.0121 (14)	−0.0044 (16)
C14	0.0608 (6)	0.0431 (6)	0.0747 (7)	0.0014 (5)	0.0019 (5)	−0.0009 (5)
C15	0.0712 (11)	0.0527 (10)	0.0963 (14)	−0.0067 (8)	−0.0075 (9)	−0.0088 (8)
C16	0.0884 (12)	0.0489 (10)	0.0970 (13)	0.0083 (8)	−0.0137 (10)	0.0082 (8)
C10	0.1033 (16)	0.0863 (14)	0.0730 (12)	0.0018 (12)	0.0032 (11)	−0.0142 (10)

### Geometric parameters (Å, º)

**Table:**

O1—C1	1.2233 (18)	C8—C12	1.516 (3)
N1—C5	1.374 (2)	C9—C10	1.375 (3)
N1—C1	1.4050 (19)	C9—H9A	0.9300
N1—C6	1.447 (2)	C11—C10	1.373 (3)
N2—C14	1.145 (2)	C11—H11A	0.9300
C1—C2	1.438 (2)	C12—C13	1.494 (4)
C2—C3	1.369 (2)	C12—H12A	0.9700
C2—C14	1.434 (2)	C12—H12B	0.9700
C3—C4	1.409 (2)	C13—H13A	0.9600
C3—C15	1.498 (2)	C13—H13B	0.9600
C4—C5	1.362 (2)	C13—H13C	0.9600
C4—H4A	0.9300	C15—H15A	0.9600
C5—C16	1.490 (2)	C15—H15B	0.9600
C6—C7	1.375 (2)	C15—H15C	0.9600
C6—C11	1.377 (3)	C16—H16A	0.9600
C7—C8	1.393 (3)	C16—H16B	0.9600
C7—H7A	0.9300	C16—H16C	0.9600
C8—C9	1.371 (3)	C10—H10A	0.9300
			
C5—N1—C1	122.98 (13)	C10—C11—H11A	120.3
C5—N1—C6	121.91 (13)	C6—C11—H11A	120.3
C1—N1—C6	115.10 (12)	C13—C12—C8	112.4 (2)
O1—C1—N1	119.87 (14)	C13—C12—H12A	109.1
O1—C1—C2	125.29 (14)	C8—C12—H12A	109.1
N1—C1—C2	114.83 (13)	C13—C12—H12B	109.1
C3—C2—C14	121.17 (14)	C8—C12—H12B	109.1
C3—C2—C1	123.00 (14)	H12A—C12—H12B	107.9
C14—C2—C1	115.82 (13)	C12—C13—H13A	109.5
C2—C3—C4	117.99 (14)	C12—C13—H13B	109.5
C2—C3—C15	121.81 (15)	H13A—C13—H13B	109.5
C4—C3—C15	120.19 (14)	C12—C13—H13C	109.5
C5—C4—C3	121.42 (14)	H13A—C13—H13C	109.5
C5—C4—H4A	119.3	H13B—C13—H13C	109.5
C3—C4—H4A	119.3	N2—C14—C2	179.11 (19)
C4—C5—N1	119.66 (14)	C3—C15—H15A	109.5
C4—C5—C16	121.84 (15)	C3—C15—H15B	109.5
N1—C5—C16	118.50 (15)	H15A—C15—H15B	109.5
C7—C6—C11	120.32 (16)	C3—C15—H15C	109.5
C7—C6—N1	120.05 (16)	H15A—C15—H15C	109.5
C11—C6—N1	119.60 (15)	H15B—C15—H15C	109.5
C6—C7—C8	120.54 (18)	C5—C16—H16A	109.5
C6—C7—H7A	119.7	C5—C16—H16B	109.5
C8—C7—H7A	119.7	H16A—C16—H16B	109.5
C9—C8—C7	118.25 (18)	C5—C16—H16C	109.5
C9—C8—C12	121.81 (19)	H16A—C16—H16C	109.5
C7—C8—C12	119.9 (2)	H16B—C16—H16C	109.5
C8—C9—C10	121.29 (19)	C11—C10—C9	120.2 (2)
C8—C9—H9A	119.4	C11—C10—H10A	119.9
C10—C9—H9A	119.4	C9—C10—H10A	119.9
C10—C11—C6	119.37 (18)		
			
C5—N1—C1—O1	176.08 (15)	C1—N1—C5—C16	−176.04 (16)
C6—N1—C1—O1	−2.4 (2)	C6—N1—C5—C16	2.3 (2)
C5—N1—C1—C2	−3.9 (2)	C5—N1—C6—C7	−85.9 (2)
C6—N1—C1—C2	177.62 (13)	C1—N1—C6—C7	92.63 (18)
O1—C1—C2—C3	−178.23 (16)	C5—N1—C6—C11	96.09 (19)
N1—C1—C2—C3	1.7 (2)	C1—N1—C6—C11	−85.41 (19)
O1—C1—C2—C14	0.4 (3)	C11—C6—C7—C8	0.9 (3)
N1—C1—C2—C14	−179.68 (14)	N1—C6—C7—C8	−177.12 (16)
C14—C2—C3—C4	−177.87 (15)	C6—C7—C8—C9	0.0 (3)
C1—C2—C3—C4	0.6 (2)	C6—C7—C8—C12	178.37 (19)
C14—C2—C3—C15	1.0 (3)	C7—C8—C9—C10	−0.7 (3)
C1—C2—C3—C15	179.52 (16)	C12—C8—C9—C10	−179.0 (2)
C2—C3—C4—C5	−1.1 (2)	C7—C6—C11—C10	−1.2 (3)
C15—C3—C4—C5	179.98 (16)	N1—C6—C11—C10	176.86 (17)
C3—C4—C5—N1	−0.9 (2)	C9—C8—C12—C13	104.2 (3)
C3—C4—C5—C16	178.71 (16)	C7—C8—C12—C13	−74.1 (3)
C1—N1—C5—C4	3.6 (2)	C6—C11—C10—C9	0.5 (3)
C6—N1—C5—C4	−178.01 (15)	C8—C9—C10—C11	0.4 (3)

### Hydrogen-bond geometry (Å, º)

**Table:**

*D*—H···*A*	*D*—H	H···*A*	*D*···*A*	*D*—H···*A*
C4—H4*A*···O1^i^	0.93	2.32	3.2105 (18)	161

Symmetry code: (i) *x*, *y*+1, *z*.
